# Powerful Complex Immunoadjuvant Based on Synergistic Effect of Combined TLR4 and NOD2 Activation Significantly Enhances Magnitude of Humoral and Cellular Adaptive Immune Responses

**DOI:** 10.1371/journal.pone.0155650

**Published:** 2016-05-17

**Authors:** Amir I. Tukhvatulin, Alina S. Dzharullaeva, Natalia M. Tukhvatulina, Dmitry V. Shcheblyakov, Maxim M. Shmarov, Inna V. Dolzhikova, Patricia Stanhope-Baker, Boris S. Naroditsky, Andrei V. Gudkov, Denis Y. Logunov, Alexander L. Gintsburg

**Affiliations:** 1 N. F. Gamaleya Research Institute for Epidemiology and Microbiology, Gamaleya str.18, 123098 Moscow, Russia; 2 Cleveland BioLabs, Inc., Buffalo, New York, United States of America; 3 Department of Cell Stress Biology, Roswell Park Cancer Institute, Elm and Carlton str., 14263 Buffalo, New York, United States of America; Dasman Diabetes Institute, KUWAIT

## Abstract

Binding of pattern recognition receptors (PRRs) by pathogen-associated molecular patterns (PAMPs) activates innate immune responses and contributes to development of adaptive immunity. Simultaneous stimulation of different types of PRRs can have synergistic immunostimulatory effects resulting in enhanced production of molecules that mediate innate immunity such as inflammatory cytokines, antimicrobial peptides, etc. Here, we evaluated the impact of combined stimulation of PRRs from different families on adaptive immunity by generating alum-based vaccine formulations with ovalbumin as a model antigen and the Toll-like receptor 4 (TLR4) agonist MPLA and the Nucleotide-binding oligomerization domain-containing protein 2 (NOD2) agonist MDP adsorbed individually or together on the alum-ovalbumin particles. Multiple *in vitro* and *in vivo* readouts of immune system activation all showed that while individual PRR agonists increased the immunogenicity of vaccines compared to alum alone, the combination of both PRR agonists was significantly more effective. Combined stimulation of TLR4 and NOD2 results in a stronger and broader transcriptional response in THP-1 cells compared to individual PRR stimulation. Immunostimulatory composition containing both PRR agonists (MPLA and MDP) in the context of the alum-based ovalbumin vaccine also enhanced uptake of vaccine particles by bone marrow derived dendritic cells (BMDCs) and promoted maturation (up-regulation of expression of CD80, CD86, MHCII) and activation (production of cytokines) of BMDCs. Finally, immunization of mice with vaccine particles containing both PRR agonists resulted in enhanced cellular immunity as indicated by increased proliferation and activation (IFN-γ production) of splenic CD4+ and CD8+ T cells following *in vitro* restimulation with ovalbumin and enhanced humoral immunity as indicated by higher titers of ovalbumin-specific IgG antibodies. These results indicate that combined stimulation of TLR4 and NOD2 receptors dramatically enhances activation of both the humoral and cellular branches of adaptive immunity and suggests that inclusion of agonists of these receptors in standard alum-based adjuvants could be used to improve the effectiveness of vaccination.

## Introduction

In addition to the target antigen, adjuvants are key components of vaccines. Adjuvants serve to (i) enhance immunogenicity of poorly immunogenic antigens, (ii) induce broader immune responses capable of covering multiple serotypes, (iii) reduce the need for booster immunizations, (iv) increase the duration of protection, and (v) allow reduction of the antigen dose needed for effective vaccination, which is financially beneficial and also reduces the risk of unfavorable side effects [1]. Despite the clear importance of adjuvant usage, research focused on their development and application has been extremely limited. In the past 70 years, only a single type of adjuvants, those based on Aluminium (Alum), has been used clinically. Alum adjuvants predominantly boost humoral immunity by providing Th2 cell help to follicular B cells [2]. This type of immune response is effective against extracellular pathogens (helminthes, *Vibrio cholera*, *Bacillus anthracis*, etc.), but not against intracellular pathogens that require mainly Th1-cell-mediated immunity (e.g., HIV, influenza virus, *Mycobacterium tuberculosis*, etc.) [3]. The facts that adjuvant development primarily involves empirical approaches and must meet strict safety requirements could explain the paucity of development activity in this area. In any case, it is reasonable to hypothesize that new adjuvants could be discovered that might have stronger and broader (e.g., stimulating both Th1- and Th2-mediated immunity) activity than Alum. Such agents could be expected to have a highly significant impact on human health through improving the effectiveness of numerous vaccines.

Discovery of the powerful immunoregulatory activities of pattern recognition receptors (PRRs) including both induction of innate immunity and modulation of adaptive immune responses suggested that agonists of PRRs could be effective adjuvants [4]. In fact, it has now been shown for a variety of PRR agonists that inclusion of the agonist as an adjuvant component in a vaccine (i) increases the magnitude of the specific immune response against the vaccine antigen, (ii) increases the longevity of the response, and (iii) steers polarization of the immune response. In particular, when administered as vaccine adjuvants, agonists of TLR-3, -4, -7/8, and -9 caused predominately Th1 response stimulation [5,6,7,8], agonists of TLR5 (e.g., flagellin) produced mixed Th1 and Th2 responses [9], and agonists of TLR2 (e.g., Pam_2_CSK_4_) showed a strong Th2-biased humoral immune response [10]. It was also shown that stimulation of a non-TLR PRR, the C-type lectin Mincle receptor, by the synthetic agonist trehalose-6,6-dibehenate (TDB) induced a strong Th1/Th17 immune response [11]. Published reports on the activity of NOD1 and NOD2 agonists are conflicting, with Th1-, Th-2, and Th17-polarization of the immune response being observed in different studies with different antigens [12,13,14], Therefore, while some details remain unclear, it is well established that number of PRR agonists can be safe and effective immunostimulatory components of vaccines. Based on this foundation, numerous PRR agonist-based adjuvants have been prepared [15]. Typically these are comprised of the PRR agonist immobilized on a particulate carrier in order to achieve better immunostimulatory characteristics. Examples of such adjuvants include: AS01 (the TLR4 agonist Monophosphoryl Lipid A (MPLA) formulated in liposomes), AS04 (MPLA adsorbed on aluminum salts), and CAF01 (the Mincle receptor agonist TDB formulated in liposomes). These adjuvants produce strong induction of adaptive immune reactions and/or more potent skewing of immune responses to different Th responses [16,17]. It has been suggested, however, that the effectiveness of these PRR-based adjuvants might be improved by using more than one PRR agonist as the immunostimulatory component within a given vaccine. The rationale for this approach is based on the demonstration of synergistic activity between members of different families of PRRs. For example, combined stimulation of different TLR and NLR family members was shown to lead to enhanced transcription factor activation and cytokine/chemokine production, providing increased protection of mice against Salmonella infection in comparison to stimulation of either receptor alone [18,19,20]. Here, we report studies aimed at evaluating the immunoadjuvant potential of combining agonists of different types of PRRs in the context of model vaccine formulations consisting of clinically approved alum salts (carrier), ovalbumin (model antigen) and the well-known PRR agonists MPLA (TLR4 agonist) and MDP (NOD2 agonist) adsorbed to alum particles alone or in combination. It is a first report showing that simultaneous combined stimulation of TLR4 and NOD2 receptors via activation of several of transcriptional factors (e.g., NF-κB, AP-1, CREB) results in drastically expand the number and increase gene expression levels in THP-1 cells in comparison to individual PRR stimulation. The PRR agonist combination also had synergistic effects on phagocytic uptake of vaccine particles by BMDCs and on dendritic cell (DC) maturation (up-regulation of CD80, CD86, and MHCII molecules) and activation (production of cytokines) *in vitro*. Most importantly, the synergistic effects of combining MPLA and MDP in the vaccine formulation extended to the cellular and humoral adaptive immune responses observed in immunized mice. These results support the possibility of significantly improving vaccine efficacy through development of new adjuvants based on synergistic PRR activity.

## Materials and Methods

### Cultured cells

Parental THP-1 cells and THP1-XBlue^™^-CD14 reporter cells (carrying an NF-κB/AP-1-inducible SEAP reporter construct) were obtained from InvivoGen (USA) and cultured in RPMI medium (GE Healthcare, USA) supplemented with 10% fetal calf serum (FCS, Thermo Scientific, USA), 50 U/ml penicillin, 50 μg/ml streptomycin, 2 mM glutamine, and 0.1 M NaHCO3 (all PanEco, Russia) at 37°C with 5% CO_2_. 200 μg/ml Zeocin, and 250 μg/ml G418 (both InvivoGen, USA) was included in the culture medium for the THP1-XBlue^™^-CD14 reporter cell line.

HEK-Blue-hTLR4, HEK-Blue-hNOD2 and control HEK-Blue-Null2 cells were obtained from Invivogen (USA) and maintained in DMEM medium (GE Healthcare, USA) supplemented with 10% fetal calf serum (Thermo scientific, USA), 50 U/ml penicillin, 50 μg/ml streptomycin, 2 mM glutamine, 0.1 M NaHCO3 (all PanEco, Russia), and 200 μg/ml Zeocin (InvivoGen, USA) at 37°C with 5% CO_2_.

### SEAP reporter assay

Reporter cells were seeded in 96-well plates at 1x10^5^ cells per well for THP-1 and THP1-XBlue^™^-CD14 cells in RPMI medium and 2x10^4^ cells per well for HEK-Blue cell lines in DMEM medium (200 μl/well). The next day, vaccine formulations were added to the wells. Eighteen hours later, secreted embryonic alkaline phosphatase (SEAP) activity was determined in the culture medium as described in [20]. Briefly, aliquots of culture medium (200 μl) were collected from each well and clarified by centrifugation at 14,000X*g* for 2 min, heated at 65°C for 5 min to inhibit endogenous phosphatase activities. Aliquots form each well (50 μl) were mixed with 150 μl with prewarmed to 37°C 1xSEAP assay buffer (0.5M carbonate, pH 9.8, 0.5mM MgCl_2_), containing 60μM *p*-nitrophenylphosphate (Sigma-Aldrich, USA). Absorbance of the reaction mixture at 405 nm was read using a Wallac 1420 spectrophotometric plate reader (PerkinElmer, USA). SEAP activity is presented in milliunits (mU) per ml. One milliunit is defined as the amount of phosphatase that hydrolyzes 1.0 pmol of *p*-nitrophenylphosphate per min.

#### Microarray-based gene expression analysis

All procedures, including sample preparation, hybridization to GeneChip Human Gene 1.0 ST Arrays (Affymetrix, USA) and data collection and analysis, were performed at LLC Bioclinicum (Russia). Briefly, parental THP-1 cells were left untreated or treated with individual PRR agonists or combinations of the agonists. Three hours later, total RNA was isolated from the cells using QIAzol reagent and purified using the RNeasy Mini Kit (all QIAGEN, USA) according to the manufacturer’s protocol. RNA integrity was analyzed using the RNA 6000 Nano Kit (Agilent, USA) and Agilent 2100 Bioanalyzer. Only samples with an RNA Integrity Number (RIN) greater than 7.5 were used for miocroarray hybridization. 0.5μg of each total RNA sample was used for cDNA synthesis using the Ambion WT Expression Kit (Thermo Fisher Scientific, USA). The obtained cDNA was labeled and hybridized to GeneChip Human Gene 1.0 ST Arrays (Affymetrix, USA) using the GeneChip^®^ WT Terminal Labeling and Controls Kit and GeneChip^®^ Hybridization, Wash, and Stain Kit (both from Affymetrix, USA) according to the manufacturer’s protocols. After hybridization and washing, arrays were scanned on an Affymetrix GeneChip 3000 7G scanner. Normalized data were converted to an expression measure for each gene on each chip using a robust modeling strategy [21]. A gene was considered induced by a particular treatment if it showed at least a 3-fold increase in expression compared to untreated cells. Two biological replicates (independently treated cell cultures) were performed for each experimental condition. The raw data have deposed at Gene Expression Omnibus (GEO) database under accession number GSE79900,

### Cytokine analysis

THP1-XBlue^™^-CD14 cells carrying an NF-κB/AP-1-dependent SEAP reporter gene or BMDCs were seeded in 96-well plates at 1x10^5^ cells per well in RPMI medium. Vaccine formulations were added to the plates on the next day (in triplicate wells). Twenty-four hours after treatment, plates were centrifuged at 1,000 rpm for 10 min, and culture supernatants were collected. Levels of 23 cytokines and chemokines (IL-1α, IL-1β, IL-2, IL-3, IL-4, IL-5, IL-6, IL-9, IL-10, IL-12 (p40), IL-12 (p70), IL-13, IL-17A, Eotaxin (CCL11), G-CSF, GM-CSF, IFN-γ, KC (CXCL1), MCP-1 (CCL2), MIP-1α (CCL3), MIP-1β (CCL4), RANTES (CCL5), and TNF-α) were measured in the prepared supernatants using the 23-plex bead-based Bio-Plex Pro kit (BioRad, USA) according to the manufacturer’s instructions.

### Phosphoprotein Profile in THP-1 cells

Human 9-plex Multi-Pathway and 6-plex NF-κB Signaling Bead Kits (EMD Millipore, Germany) were used to detect changes in phosphorylated ERK/MAP kinase 1/2 (Thr185/Tyr187), Akt (Ser473), STAT3 (Ser727), JNK (Thr183/Tyr185), p70 S6 kinase (Thr412), NF-κB (Ser536), STAT5A/B (Tyr694/699), CREB (Ser133), p38 (Thr180/Tyr182), NF-κB (Ser536), FADD (Ser194), IKKα/β (Ser177/Ser181) and IκB (Ser32), as well as total protein levels of TNFR1 and c-Myc, in THP-1 cell lysates using fluorescent bead-based immunoassay technology and Luminex system. Prior to the experiment, THP-1 cells were serum-starved (2% FCS in RPMI medium) for 24 hours and then seeded in 48-well plates at 4x105 cells per well. Twenty-four hours later, the cells were treated with MDP (20 μg/ml) and MPLA (1 μg/ml) individually or in combination. Intact (untreated) THP-1 cells were used as a control. Twenty minutes after addition of the PRR agonists, the cells were harvested, washed with PBS and lysed in MILLIPLEX MAP lysis buffer in the presence of Protease Inhibitor Cocktail Set III (Calbiochem, Germany). To remove particulate matter, lysates were centrifuged at 10,000Xg for 5 min. The total protein concentration of each clarified lysate was normalized by dilution in Assay Buffer in 25μl (10 μg total protein/well). All manipulations were done on ice or at 4°C. Samples were analyzed according to the manufacturer’s instructions immediately after preparation as described above. Mean Fluorescence Intensity (MFI) of each sample was measured with the Bio-Plex MAGPIX multiplex reader (Bio-Rad, USA).

### Vaccines and adjuvants

To obtain alum-adsorbed vaccine formulations, a solution of alum salts (6 mg/ml) (SPI Pharma,USA) was mixed on a shaker (300 rpm) for 30 min at 25°C in 10 mM Tris-HCl pH7.4, 0.9% NaCl with the model antigen, endotoxin-free ovalbumin (Sigma-Aldrich, USA) (10mg/dose) alone or with immunostimulatory molecules Monophosphoryl Lipid A (MPLA) (1 μg /dose) (Sigma-Aldrich, USA) and muramyl dipeptide (MDP) (20 μg /dose) (Invitrogen, USA) individually or in combination. The volume of each formulation was adjusted with 10 mM Tris-buffer to a total of 200 μl/dose. The formulations were kept at room temperature for 30 min with intermittent mixing before use.

Average particle size, Polydispersity Index (PDI) and zeta-potential measurements were done on a Malvern Zetasizer Nano instrument (Malvern Instruments, UK) and analyzed with Zetasizer 7.01 software (Malvern Instruments, UK). Average particle size and Polydispersity Index (PDI) were determined by the dynamic light scattering method at a 10× dilution in 10 mM Tris-buffer pH 7.4 in a UV micro-cuvette (BrandTech, USA). The zeta-potential (laser-Dopplerelectrophoresis) of vaccine formulations and individual components was analyzed at a 200× dilution in 10 mM Tris-buffer pH 7.4 in folded capillary cells (Malvern Instruments, UK). Adsorption efficacy of ovalbumin to alum particles was measured by ELISA using self-made mouse anti-ovalbumin polyclonal antibodies and an ovalbumin standard calibration curve. For evaluation of adsorption efficacy of PRR agonists on Alum particles, samples were centrifugated at 10,000Xg for 5 min. The obtained supernatants containing soluble unbound PRR agonists were used for stimulation of HEK-Blue-hTLR4 and HEK-Blue-hNOD2 reporter cells containing an NF-κB/Ap-1-dependent SEAP reporter construct and artificially expressing hTLR4 and hNOD2, respectively (all Invivogen, USA). Parental HEK-Blue-Null2 reporter cells were used as a negative control.

### Mice

All animal experiments used inbred female C57BL/6 mice weighing 18 to 20 g that were purchased from the Pushchino Nursery (Institute of Bioorganic Chemistry of the Russian Academy of Sciences, Pushchino, Russia). The mice were fed a complete pelleted laboratory chow and had access to food and tap water *ad libitum*. All of the experimental procedures conform to the Guide for the Care and Use of Laboratory Animals published by the National Institutes of Health (NIH Publication #85–23, revised 1996), and approved by Institutional Animal Care and Use Committee of N.F.Gamaleya Research Center for Epidemiology and Microbiology.

### Immunizations

Female C57BL/6 mice were given two s.c. injections of vaccine formulations at the base of the tail with a two-week interval between the injections (10 mice per group). Five mice from each group were bled 14 days after the last immunization for IgG antibody titer evaluation and subsequently euthanized by CO_2_ overdose. At the same time point, the other five animals from each group were euthanized by CO_2_ overdose and their spleens were collected for analysis of T-cell responses.

### Dendritic cell cultures

Bone marrow derived dendritic cells (BMDCs) from C57BL/6 mice were differentiated from proliferating mouse bone marrow progenitors through induction with 20 ng/ml granulocyte macrophage colony stimulating factor (GM-CSF) (R&D Systems, USA) over 6–9 days as described [22]. Briefly, mice were euthanized by CO_2_ overdose and the femurs and tibias were collected in ice-cold Hank’s balanced salt solution (HBSS, Sigma-Aldrich, USA). The muscles were removed with a scalpel and by rubbing the bones with a tissue. The ends of the bones were then cut off with scissors and crushed. The bone marrow was flushed out with 2–3 ml of RPMI complete medium in a syringe with a 25-gauge needle. All bone marrow cells were collected and washed twice with HBSS. The bone marrow cells were cultured in 24-well plates containing approximately 5 × 10^5^ cells/ml in 1 ml total volume. The cells were maintained at 37°C with 5% CO_2_ in complete RPMI medium with 10% heat inactivated fetal calf serum (PAA), 0.05mM mercaptoethanol (Thermo Fisher Scientific, USA), Non Essential Amino Acids (PanEco, Russia), 20ng/ml GM-CSF, 2 mM glutamine, 100 U/ml penicillin, and 100 μg/ml streptomycin (all PanEco, Russia). After 24 hours, the nonadherent cells were collected and discarded and 1 ml of fresh media was added to each well. On day 3,5 and 7 half of the medium in each well was replaced with fresh medium. On day 7–9, the percentage of CD11c-positive cells in non-adherent population in the cultures was ~60–70%.

### Phagocytosis assay

To assess phagocytosis of vaccine particles by BMDCs, ovalbumin was labeled using a FITC labelling kit (Thermo Scientific, USA) and a pHrodo Red Microscale Labeling Kit (Life Technologies, USA) according to the manufacturer’s instructions. The pH-sensitive rhodamine-based pHrodo Red dye is non-fluorescent at neutral pH, but turns bright red upon acidification. Because it is both fluorogenic and pH-sensitive, the pHrodo Red dye can be used as a specific sensor of acidification of the phagosome following phagocytosis. This allows for reliable distinction between internalized particles and particles that are simply cell-associated.

The labelled ovalbumin was extensively washed and the labeling efficiency was checked using a Synergy H4 hybrid reader (Bio-Tek, Germany) according to the manufacturer’s protocols. The degree of labeling was 5 for pHrodo and the final Fluorescein/Protein Molar Ratio was 2 for FITC. The labelled ovalbumin was then used for preparation of vaccine formulations as described above for unmodified ovalbumin.

Bone Marrow-derived Dendritic Cells (BMDCs, see above for preparation) were cultured in 24-well plates on 13-mm glass coverslips in complete RPMI medium. Forty minutes after addition of vaccine formulations, cells were washed twice with ice-cold HBSS and live cells were immediately analyzed by fluorescence microscopy using a Z1 Imager microscope (Carl-Zeiss, Germany) with x40 optics and G365, 470/40nm and 546/12 nm filter sets for DAPI, FITC and pHrodo fluorescence detection, respectively.

For flow cytometric analysis, cells were collected using trypsin*-*EDTA solution and analyzed on a FACS AriaIII instrument (BD biosciences, USA).

### Flow cytometric analysis of BMDCs

BMDCs were seeded in 24-well plates at 2x10^5^cells per well in RPMI complete medium (with 10% FCS). Vaccine formulations containing MPLA and MDP, individual PRR agonists or no PRR agonist (Alum+ovalbumin alone) were added to the cells and 24 hours later, the cells were stained with antibodies for FACS analysis. Additional controls were BMDCs treated with medium alone or with soluble ovalbumin (no alum or PRR agonist). Cells were stained with fluorescently labeled anti-CD11c PE-CF594 (clone HL3), anti-CD80 PE (clone 16-10A1), anti-CD86 AF700 (clone GL1), and anti-MHCII PE (clone 2G9) monoclonal antibodies or the corresponding isotype controls for 20 min at 4°C in Staining Buffer (all, BD biosciences, USA), fixed in 1% paraformaldehyde, and then stored at 4°C until analysis on a FACS AriaIII instrument (BD biosciences, USA).

### Analysis of T cell responses

Mice were euthanized 14 days after the second immunization and splenocytes were isolated from harvested spleens using Falcon 70μm nylon mesh filter and purified by Ficoll 1.09 g/mL (PanEco, Russia) density gradient centrifugation (400Xg, 30 min). Antigen-specific T cell responses were measured by CFSE T Cell Proliferation and intracellular IFNγ staining methods. For analysis of T cell proliferation, splenocytes were stained with Carboxyfluorescein succinimidyl ester (CFSE) tracer kit (Invitrogen, USA) as described previously [23] and seeded in 96-well plates at 3 x 10^5^ cells per well. Cells were restimulated with whole ovalbumin antigen at 1μg/ml and cultured in complete RPMI medium at 37°C in 5% CO2. 72 hours later, cells were harvested, washed in PBS and stained with anti-CD3 APC, anti-CD8 APC-Cy7 and anti-CD4 PE-CF594 for 20 min at 4°C, in Staining Buffer (all, BD biosciences, USA), fixed in 1% paraformaldehyde, then stored at 4°C until analysis. Flow cytometric analysis was performed on a FACSAriaIII (BD Biosciences, USA) flow cytometer with BD FACSDiva Software (BD Biosciences, USA). Proliferating CD4 or CD8 T lymphocytes were identified by forward and side light scatter, expression of CD3, CD4, CD8 and low fluorescence intensity of CFSE dye. For intracellular IFN-γ staining, splenocytes were seeded in 24-well plates at 1 x 10^6^ cells per well and restimulated for 18 hours with whole ovalbumin antigen at 1μg/ml in the presence of BD GolgiPlug solution (BD biosciences, USA). Cells were then labeled with anti-CD3 APC (clone 145-2C11), anti-CD4 PE-CF594 (clone RM4-5) and anti-CD8 APC-Cy7 (clone 53–6.7) or the corresponding isotype controls, fixed using the Cytofix/Cytoperm kit, and washed with Perm/Wash buffer before labeling with anti-IFN PE-Cy7 (clone XMG1.2) (all, BD Biosciences, USA). Flow cytometric analysis was performed as described above for the CFSE proliferation assay.

### Measurement of antigen-specific antibody titers in serum samples from immunized mice

Total ovalbumin-specific IgG and IgG isotypes titers were measured in serum samples prepared from blood collected from the mice 14 days after the second immunization. Briefly, 96-well microtiter Immuno plates (SPL, South Korea) were coated with 100 μl per well of 10 μg/ml ovalbumin solution in coating buffer (137 mM NaCl, 2.7 mM KCl, 8.1 mM Na_2_HPO_4_, and 1.5 mM KH_2_PO_4_) and incubated overnight at 4°C. On the next day, plates were washed three times with PBS containing 0.05% Tween20 (PBS-T) and blocked in PBS-T with 3% nonfat milk (Sigma-Aldrich) for 1 hour at 37°C. Serum samples (100μl per well) were then added to the coated, washed and blocked ELISA plates in serial 2-fold dilutions starting from 1:1250 and ending at 2,560,000. Serum samples were diluted using PBS-T. After incubating the plates with sera for 1 hour at 37°C, the plates were washed three times with PBS-T and 100 μl horseradish peroxidase-labelled secondary antibody was added to each well. Secondary antibodies were goat anti-mouse total IgG (GE Healthcare, Germany) (diluted 1:5000 in PBS-T) and goat anti-mouse IgG1, IgG2a IgG2b, or IgG3 (Abcam, UK) (diluted 1:5000 in PBS-T). The plates were incubated again for 1 hour at 37°C and then washed three times with PBS-T. For detection of bound antibodies, 100 μl of the peroxidase substrate 3,3′,5,5′-Tetramethylbenzidine (TMB) was added to each well. The plates were incubated at 25°C for 20 min for color development. The reaction was stopped using 4M H_2_SO_4_ and the optical density in each well was measured at 450nm using a Multiscan FC spectrophotometric plate reader (Thermo Fisher, USA).

### Statistical analysis

All experiments were performed three times (each in triplicate) unless otherwise specified and data are expressed as the mean ± SD of the values from all experiments. Statistical significance was assessed using a two-tailed unpaired Student’s t-test with a threshold set at p < 0.05.

## Results

### Combined treatment of THP1 cells with agonists of TLR4 and NOD2 leads to a stronger and broader transcriptional response than treatment with either single agonist

It has been demonstrated that simultaneous activation of TLR and NOD family receptors has a synergistic effect on NF-κB activation and production of pro-inflammatory cytokines and chemokines (IL-1β, IL-8, TNF-α, MIP-1α, etc.) both *in vitro* and *in vivo* [18,19,20]. However, it is possible that combined stimulation of TLR and NOD receptors has broader effects on gene expression that could contribute to adjuvant activity. Here we tested this hypothesis by using microarray-based global gene expression analysis to compare the transcriptional profiles of naïve THP-1 cells versus THP-1 cells treated with the TLR4 agonist MPLA and the NOD2 agonist MDP either alone or in combination.

First, in order to determine the optimal doses of MPLA and MDP to use in the transcriptional profiling experiment (i.e., doses that produce synergy upon combination of the two agonists), we treated THP1-XBlue^™^-CD14 cells carrying an NF-κB/AP-1-dependent SEAP reporter gene with different doses of the two agonists alone and in combination for 18 hours and then measured activity of expressed SEAP as a readout of NF-κB/AP-1 activation. Based on the obtained data, we chose 20μg/ml MDP and 1μg/ml MPLA for use in the subsequent experiments as a combined regimen that provides synergistic NF-κB/AP-1 stimulation in THP1 cells over either agonist alone (S1 Fig).

**Fig 1:**
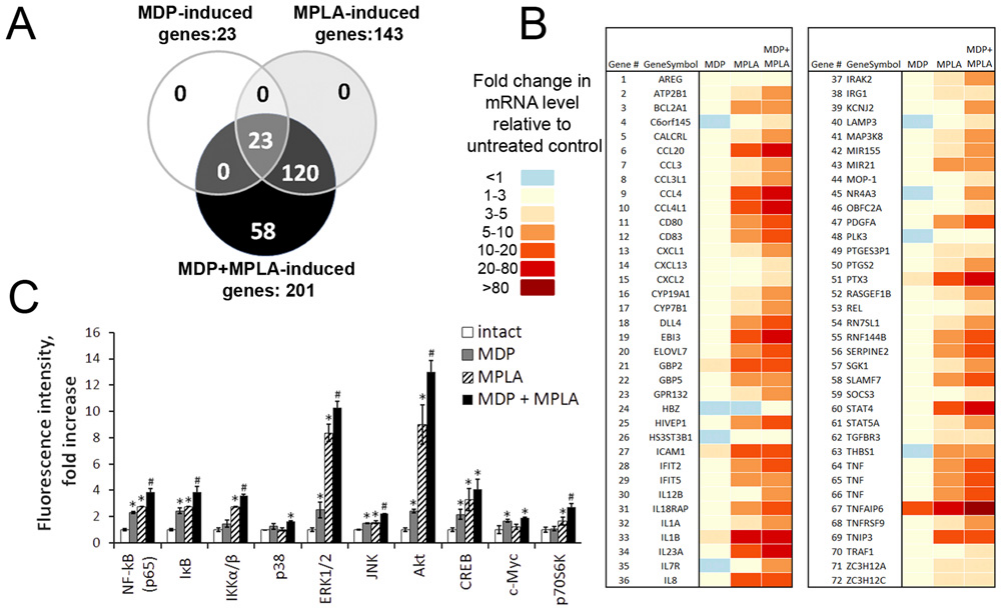
Combination of MPLA and MDP strongly enhances the transcriptional response in THP-1 cells in comparison to individual PRR agonists. **(A)** Venn diagram demonstrating overlap in the sets of genes found to be MDP-, MPLA- and/or MDP+MPLA-induced in THP-1 cells. Cells were treated with MDP (20μg/ml) and MPLA (1μg/ml) individually or in combination or left unstimulated. RNA was harvested after 3 h and analyzed by hybridization to Affymetrix Human Gene 1.0 ST genechip microarrays. Genes induced by MDP (white circle), MPLA (gray circle) or their combination (black circle) by at least 3-fold over untreated cells are shown. **(B)** List of MDP+MPLA-induced genes with potentiated expression in comparison to gene expression levels in untreated cells and cells treated with either MPLA or MDP alone. Cells were left untreated or treated with MDP (20μg/ml) and MPLA (1μg/ml) individually or in combination. RNA was harvested after 3 h and analyzed by hybridization to Affymetrix Human Gene 1.0 ST Genechip microarrays. The figure lists 72 genes that showed potentiated expression with MDP+MPLA treatment compared to MPLA or MDP alone (potentiated = fold induction of gene expression level with combined treatment over the untreated control was greater than the sum of the fold induction seen with MPLA alone and MDP alone). Statistical significance was determined by a random-variance t-test with P<0.01 as a cut-off for signficance. The magnitude of the fold-change in gene expression relative to untreated cells is indicated by color. **(C)** Multiplex analysis of molecular pathways activated via phosphorylation in THP-1 cells in response to individual or combined TLR4 and NOD2 stimulation. Cells were left untreated or treated for 20 min with MDP (20 μg/ml) and MPLA (1 μg/ml) alone or in combination. Phosphoprotein levels of p65 subunit NF-κB (Ser536), IκB (Ser32), IKKα/β (Ser177/Ser181), p38 (Thr180/Tyr182), ERK/MAP kinase 1/2 (Thr185/Tyr187), JNK (Thr183/Tyr185), Akt (Ser473), CREB (Ser133), STAT3 (Ser727), p70 S6 kinase (Thr412), and STAT5A/B (Tyr694/699), as well as total protein levels of TNFR1 and c-Myc were evaluated using MILLIPLEX Magnetic Bead Signaling kits and a Bio-Plex MAGPIX multiplex reader (Bio-Rad). Data was collected from triplicate samples in two independent experiments and is presented as mean fluorescent intensity (MFI) ± SD. * indicates significant difference (P≤0.05) between formulations containing MDP or MPLA individually and the formulation without PRR agonists. # indicates significant difference (P≤0.05) between group treated with Alum+OVA+MDP+MPLA and Alum+OVA+MPLA or Alum+OVA+MDP (Student’s t-test).

Therefore, for the transcriptional profiling experiment, THP1 cells were treated with 1μg/ml MPLA and 20μg/ml MDP individually or in combination. RNA for microarray hybridization was harvested from the cells 3 hours after treatment. The data from this experiment (see Methods for details) showed that treatment with MPLA led to induction (by at least 3-fold compared to untreated THP1 cells) of 143 genes, whereas treatment with MDP only led to induction of 23 genes (Fig 1A). Treatment of THP1 cells with these two agonists in combination resulted in induction of 201 genes. Importantly, all of the genes that were induced by individual treatment with MDP or MPLA were also induced by combined treatment with both agonists. The 58 additional genes that were identified as induced by combination treatment but not by single agonist treatment were all genes that were expressed in the single agonist-treated cells, but did not meet the level of induction (3-fold relative to untreated cells) used as a cut-off. It is worth noting that the level of expression of the majority of genes induced (relative to untreated cells) by combined TLR4/NOD2 stimulation was significantly greater than that observed in cells treated with the agonist of one receptor or the other. Genes with “potentiated” expression were defined as those for which the fold induction of gene expression after combined stimulation of TLR4 and NOD2 relative to untreated cells was greater than the sum of the fold induction observed after stimulation of either TLR4 or NOD2 alone. Seventy-two genes showed potentiated expression with combination of the TLR4 and NOD2 agonists (Fig 1B). Most of these genes are known to be involved in immediate innate immune reactions (e.g., encoding proinflammatory cytokines, molecules involved in arachidonic acid metabolism, antimicrobial peptides, etc.), while others are markers of monocyte maturation and differentiation that may reflect rather than mediate the induced adaptive immune response (e.g. CD54, 80, 83, LAMP3). Potentiated expression of representative genes (*Il1β*,*Il8*,*Tnf*) after combined stimulation of TLR4 and NOD2 in THP-1 cells was confirmed by measuring levels of the encoded cytokines in cell culture supernatants (S2 Fig). Therefore, by applying a 3-fold cut-off for induction, combined stimulation of TLR4 and NOD2 was found to expand the repertoire (number) of induced genes and also produce substantially higher gene expression levels (greater than additive) in comparison to cells treated with agonists of one receptor or the other. To investigate the molecular mechanisms responsible for potentiated gene expression after combined activation of TLR4 and NOD2 receptors in THP1 cells, we conducted a protein phosphorylation profiling of several signaling kinases and transcription factors in THP-1 cells upon individual and combined stimulation of TLR4 and NOD2 receptors using Milliplex Magnetic Bead assay kits.

Stimulation of TLR4 by MPLA treatment for 20 minutes led to phosphorylation of a larger number of different proteins and higher levels of phosphorylation than observed with NOD2 stimulation (Fig 1C). For example, IKK (2.7-fold increase over untreated cells) and p70S6K (2.7-fold increase) became phosphorylated after TLR4 activation but not after NOD2 activation. Proteins that were phosphorylated following either single treatment but showed a greater level of phosphorylation after TLR4 stimulation compared to NOD2 stimulation included p65 (2.8-fold increase over untreated cells with TLR4 agonist versus 2.3-fold with NOD2 agonist), IκB (2.8-fold versus 2.4-fold), ERK (8.4-fold versus 2.5-fold), Akt (9.0-fold versus 2.4-fold) and CREB (3.3-fold versus 2.1-fold). Combined stimulation of TLR4 and NOD2 had an even stronger effect on these signaling pathways, resulting in increased phosphorylation of p65 (3.8-fold increase over untreated cells), IκB (3.8-fold increase), IKK (3.6-fold increase), ERK (10.3-fold increase), Akt (13.2-fold increase), CREB (4.1-fold increase), and p70S6K (2.7-fold increase) as well as increased protein levels of c-Myc (1.9-fold over untreated cells). Thus, all proteins that showed increased phosphorylation with single stimulation of either TLR4 or NOD2 were also affected (and to a greater extent) by combined stimulation of both PRRs. Phosphorylation of FADD (Ser194), STAT3 (Ser727) and STAT5A/B (Tyr694/699) and elevated expression of TNFR1 was not observed with either single or combined stimulation of TLR4 and NOD2. This was somewhat unexpected, but likely reflects the influence of specific aspects of this experiment such as cell type, treatment time or kit sensitivity [24,25,26,27].

Overall, these data demonstrate synergy between TLR4 and NOD2 in stimulating several signaling pathways including those mediated by NF-κB-, AP-1- (predominantly via ERK kinase), p70S6K, and Akt/CREB. These pathways are known to regulate numerous important cell functions, including cell survival, proliferation and differentiation, and development and regulation of immune responses. Our identification of affected signaling pathways and broad spectrum of genes with potentiated expression indicates that synergism between TLR4 and NOD2 is not limited to immediate innate immune reactions mediated by potentiated activity of transcription factors NF-κB and AP-1.

### Vaccine formulations including a combination of TLR4 and NOD2 agonists adsorbed on alum particles induce synergistic activation of NF-κB/AP-1 pathways in vitro

Based on the observed synergistic effects of combined TLR4 and NOD2 stimulation described above, next we hypothesized that immunoadjuvant properties of Alum that is routinely used in vaccine formulations, can be enhanced by adding properly rationed TLR4 and NOD2 agonists, combination of which demonstrated synergy in NF-κB stimulation in above described experiments. The negative charge of free ovalbumin (-10.9 ± 5.42 mV), MPLA (-45.5 ± 9.73mV) and MDP (-25.8 ± 4.22 mV) and the positive charge of alum particles (+26.8 ± 1.42 mV) under neutral pH conditions makes it possible to create stable complexes in which the antigen and PRR agonist(s) were non-covalently bound to alum. However, use of antigen or PRR agonists in doses exceeding the binding capacity of the alum salts, leads to instability of the formulations evidenced by a decrease in zeta potential (up to 0 mV) and aggregation of particles (S3 Fig). To ensure stability of our vaccine formulations, we used ratios of antigen, PRR agonists and alum that would not utilize the full alum binding capacity. The generated vaccine formulations composed of Alum+ OVA (control with no PRR agoinist), Alum+OVA+MDP, Alum+OVA+MPLA, Alum+OVA+MDP+MPLA are shown in Table 1. Their physical characteristics: average particle size, polydispersity index (PDI), and zeta-potential are listed in S1 Table. All three of these characteristics remained unchanged over a 4-week period, demonstrating the high stability of the formulations (data not shown).

**Table 1:** Composition of prepared model vaccine formulations.

Group	Dose of Alum (μg per dose)	Dose of ovalbumin (μg per dose)	Immunostimulatory molecules (μg per dose)	
			MDP	MPLA
**Alum + OVA**	**600**	**10**	**-**	**-**
**Alum + OVA + MDP**	**600**	**10**	**20**	**-**
**Alum + OVA + MPLA**	**600**	**10**	**-**	**1**
**Alum + OVA + MDP + MPLA**	**600**	**10**	**20**	**1**

**Fig 2:**
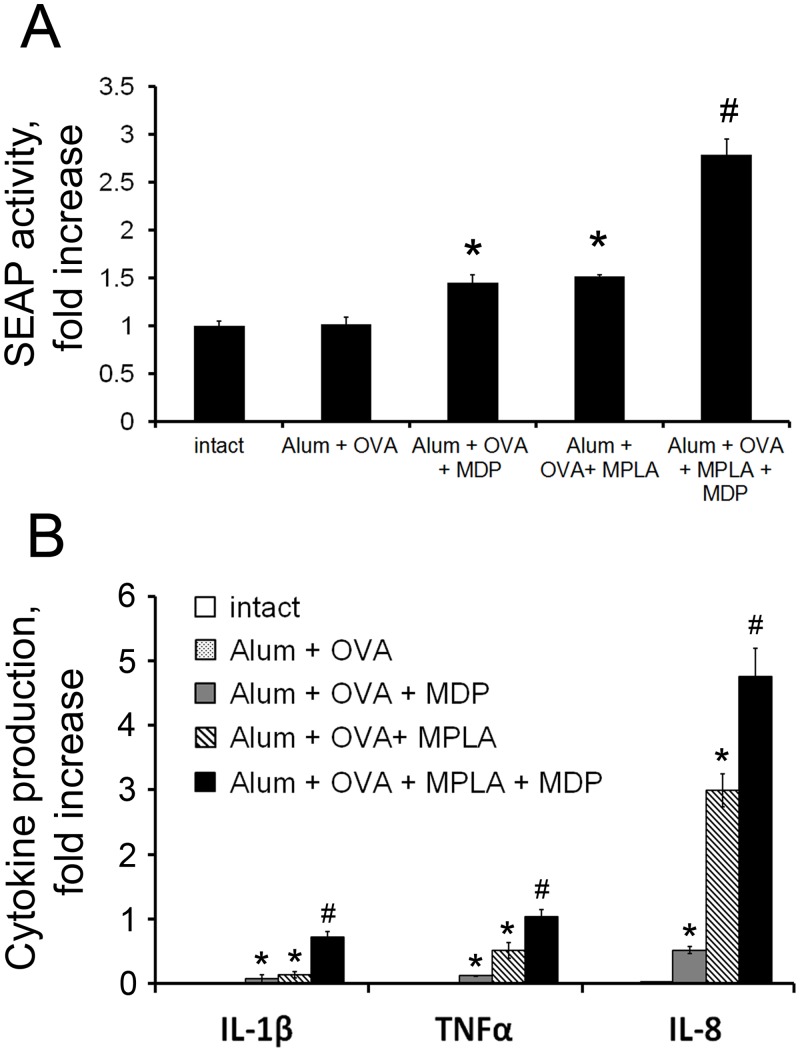
Vaccine formulations containing a combination of TLR4 and NOD2 agonists enhance NF-κB/AP-1 activation in THP-1 cells compared to formulations containing individual agonists. **(A)** Addition of the vaccine formulation containing both TLR4 and NOD2 agonists to THP1-XBlue^™^-CD14 cells leads to enhanced NF-κB/AP-1-dependent SEAP activity compared to formulations with individual PRR agonists. SEAP activity was measured in cell-free culture supernatants 18 h after vaccine formulation addition. Results are expressed as the fold-increase in SEAP activity relative to untreated (intact) cells; mean values ± SD from three independent experiments, each performed in duplicate. * indicates significant difference (P≤0.05) between formulations containing MDP or MPLA individually and the formulation without PRR agonists. # indicates significant difference (P≤0.05) between Alum+OVA+MDP+MPLA treatment and Alum+OVA+MPLA or Alum+OVA+MDP (Student’s t-test). **(B)** Addition of the vaccine formulation containing both TLR4 and NOD2 agonists to THP1 cells leads to enhanced cytokine production in comparison to vaccine formulations with individual PRR agonists. Cells were left untreated or treated with Alum+OVA, Alum+OVA+MDP, Alum+OVA+MPLA, or Alum+OVA+MDP+MPLA formulations for 18 hrs. Cell-free supernatants were prepared and analyzed by multiplex-bead ELISA Bio-Plex Pro kit (BioRad, USA) for production of IL-1β, TNF-α, and IL-8. Results are representative of two separate experiments, each performed in triplicate. Mean ± SD is shown for triplicate samples. * and # indicate significant differences as described for (A).

To confirm that the synergistic effects of combined TLR4 and NOD2 stimulation were not affected by adsorption of the PRR agonists on alum, we tested the NF-κB/AP-1 stimulating capacity of the prepared vaccine formulations in THP1-XBlue^™^-CD14 reporter cells (as done earlier with free PRR agonists, see above and S1 Fig). We used fixed-dose vaccine formulations containing concentration of individual PRR agonists (and ratio of PRR agonists for Alum+OVA+MDP+MPLA formulation), showed synergistic NF-κB/AP-1 stimulation when PRR agonists were added in soluble forms. NF- κB/AP-1-dependent SEAP activity showed 1.01-fold, 1.44-fold, 1.51-fold, and 2.78-fold activation (relative to untreated cells) in cells treated with Alum+OVA, Alum+OVA+MDP, Alum+OVA+MPLA, and Alum+OVA+MDP+MPLA vaccine formulations, respectively (Fig 2A). This shows that synergy between MDP and MPLA is preserved when they are adsorbed on alum within a vaccine formulation.

We next determined the effect of inclusion of PRR agonists in vaccine formulations on production of downstream effectors, such as proinflammatory cytokines. THP1 cells were treated with the different vaccine formulations and production of IL-1β, TNFα and IL-8 were examined 18h later using a Bio-plex kit. Similar to what was observed for NF-κB/AP-1-dependent SEAP expression in the THP1-XBlue^™^-CD14 reporter cells, a clear synergistic (greater than additive) response was observed in production of IL-1β, TNFα and IL-8 after treatment of THP1 cells with the Alum+OVA+MDP+MPLA formulation compared to those containing only one of the PRR agonists (Fig 2B).

Taken together, these results indicate that vaccine formulations containing TLR4 and NOD2 agonists stably absorbed on alum particles retain the activity of the PRR agonists, including their synergistic effects, and could therefore be more effective for immunization than formulations containing only alum as the adjuvant.

### Vaccine particles containing a combination of TLR4 and NOD2 agonists stimulate phagocytosis of particles by BMDCs more than particles containing individual PRR agonists

Inclusion of MPLA in vaccine formulations along with Alum and a test antigen was shown to increase local NF-κB activation and cytokine production at the site of injection and increase activation of BMDCs resulting in increased antigen uptake (phagocytosis) by these key antigen presenting cells [28]. Here, we evaluated the effect of inclusion of both TLR4 and NOD2 agonists in vaccine formulations on the phagocytosis efficiency of BMDC. For this experiment, we generated Alum+OVA, Alum+OVA+MDP, Alum+OVA+MPLA, and Alum+OVA+MDP+MPLA particles using ovalbumin that was labeled with FITC and pH-dependent pHrodo dyes. BMDCs were incubated with vaccine formulations for 40 minutes, washed extensively, and then the degree of vaccine particle internalization was determined by fluorescence microscopy (showing cellular location of particles) and flow cytometry. Use of the pH-dependent pHRodo dye allowed to differentiate between internalization of particles and their adhesion to the external cell surface. As described in the Methods section, internalized vaccine particles are specifically detected by pHrodo fluorescence (Red fluorescence) while FITC fluorescence shows both internalized and surface-bound particles and corresponds to quantity of vaccine particles added to the cells in total.

**Fig 3:**
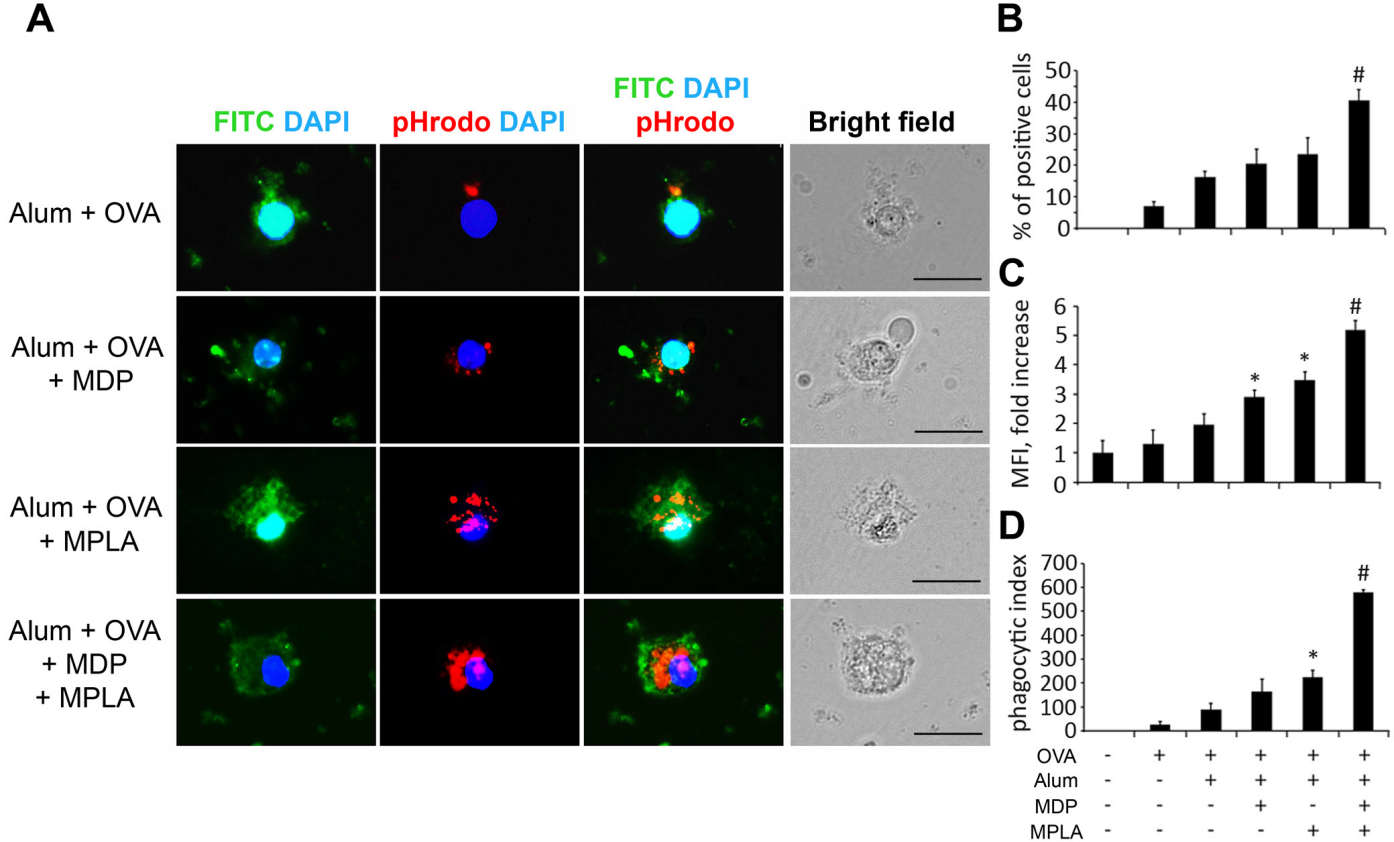
Vaccine formulations containing a combination of TLR4 and NOD2 agonists enhance the phagocytic activity of BMDCs compared to formulations containing individual agonists. Immature BMDCs isolated from naïve C57BL/6 mice were treated with Alum+OVA, Alum+OVA+MDP, Alum+OVA+MPLA and Alum+OVA+MDP+MPLA formulations produced with ovalbumin labeled with FITC and pHRodo dye for 40 min. Cells were then washed with PBS twice and evaluated by fluorescent microscopy and flow cytometry. **(A)** Representative fluorescence microscopy images of BMDCs treated with vaccine formulations. Each column shows images obtained with different filters to visualize FITC (green), pHRodo (red) and DAPI (blue) fluorescence. Phagocytosed particles are specifically visualized by pHRodo (red) fluorescence, whereas total (Extra- and intracellular) quantity of vaccine particles show FITC fluorescence. Images with all three fluorescence signals merged and bright field images also are shown. Scale bar represents 20μm. **(B)** % of cells that were pHRodo-positive; (C) fold-increase in mean fluorescence intensity (MFI) relative to untreated BMDCs; and (D) phagocytic index calculated by multiplying the percentage of pHRodo-positive cells by the MFI. Each bar represents the mean ± SD from at least three independent experiments, each performed with triplicates. Statistically significant differences were determined by Student’s t-test with P≤0.05 cut-off. * indicates significant difference (P≤0.05) between formulations containing MDP or MPLA individually and the formulation without PRR agonists. # indicates significant difference (P≤0.05) between Alum+OVA+MDP+MPLA treatment and Alum+OVA+MPLA or Alum+OVA+MDP (Student’s t-test).

After 40 minutes of incubation with BMDCs, vaccine particles containing no PRR agonists (Alum+OVA) were predominantly found on the extracellular surface of the BMDCs (green signal), with only a small amount in the cytoplasm (red signal) (Fig 3A). Inclusion of MDP or MPLA individually in the vaccine formulations resulted in increased particle internalization as visualized by red fluorescence. Even higher levels of internalization were observed with the vaccine formulation containing both PRR ligands. No fluorescence (either green or red) was detected in BMDCs incubated with vaccine formulations containing unlabeled OVA (data not shown).

Consistent with a previous study [29], flow cytometric analysis of BMDCs incubated with different vaccine formulations containing FITC- and pHrodo-labeled OVA revealed that compared to soluble antigen, physical adsorption of OVA on alum particles resulted in increased antigen phagocytosis by BMDCs (16,3% versus 7,1% for soluble antigen) (Fig 3B). Compared to Alum+OVA inclusion of individual MDP or MPLA in model vaccine formulation resulted in greater antigen internalization (20.5% and 23.6% of BMDCs were pHrodo-positive, respectively, compared to 16.3% with Alum+OVA). The strongest effect was observed when BMDCs were incubated with vaccine formulation containing both MDP and MPLA (40.7% of cells were pHrodo-positive). In addition to affecting the proportion of cells with internalized vaccine particles, the number of internalized particles per cell (indicated by mean fluorescence intensity, MFI) was increased in a similar manner by inclusion of individual or combined PRR agonists in the vaccine formulations. Thus, compared to Alum+OVA, vaccine formulations with PRR agonists produced higher MFI values, particularly if both MDP and MPLA were included in the same formulation (1.5-, 1.8- and 2.7-fold over Alum+OVA for Alum+OVA+MDP, Alum+OVA+MPLA, and Alum+OVA+MDP+MPLA, respectively; Fig 3C). These differences were reflected in the phagocytic index, calculated by multiplying the MFI (which corresponds to approximate average number of particles phagocytized per cell) by the percentage of pHrodo-positive cells (Fig 3D). Compared to incubation of BMDCs with Alum+OVA (no PRR agonist), Alum+OVA+MDP, Alum+OVA+MPLA and Alum+OVA+MDP+MPLA formulations resulted in increases in phagocytic index of 1.9-fold, 2.5-fold and 6.6-fold, respectively.

Therefore, this study is the first report demonstrating that inclusion of agonists of PRR receptors from different families in Alum-based vaccine formulations results in enhanced levels of vaccine particle phagocytosis by BMDCs compared to use of Alum alone or Alum with a single PRR agonist.

### Vaccine formulations containing a combination of TLR4 and NOD2 agonists significantly enhance maturation of BMDC compared to formulations containing individual PRR agonists

**Fig 4:**
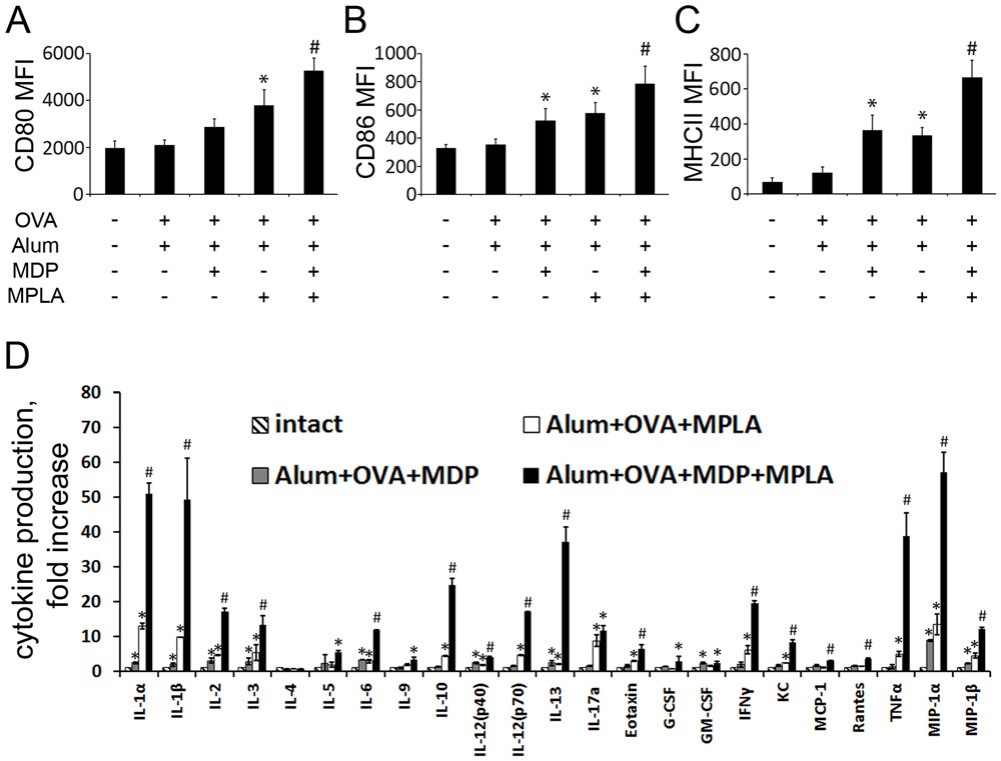
Vaccine formulations containing a combination of TLR4 and NOD2 agonists significantly enhance maturation of BMDC compared to formulations containing individual PRR agonists. BMDCs were harvested on day 8 of culture and incubated in 24-well plates for 24 hours with vaccine formulations. Expression of the maturation markers, CD80 **(A)**, CD86 **(B)**, and major histocompatibility complex (MHC) class II **(C)** was assessed by flow cytometric analysis of 5x10^4^ CD11c+ cells. Mean MFI values (indicating expression level) ± SEM from two independent experiments with 5 replicates each are shown. * indicates significant difference (P≤0.05) between formulations containing MDP or MPLA individually and the formulation without PRR agonists. # indicates significant difference (P≤0.05) between Alum+OVA+MDP+MPLA treatment and Alum+OVA+MPLA or Alum+OVA+MDP (Student’s t-test). **(D)** Cytokine levels were measured in cell-free culture supernatants collected 24 hours after addition of vaccine formulations to BMDCs (4x10^4^ cells/well) using bead-based immunoassay. Data represent mean ± SD. * indicates significant difference (P≤0.05) between formulations containing MDP or MPLA individually and the formulation without PRR agonists. # indicates significant difference (P≤0.05) between Alum+OVA+MDP+MPLA treatment and Alum+OVA+MPLA or Alum+OVA+MDP (Student’s t-test).

It is known that TLR4 and NOD2 signaling can regulate the maturation of DCs involving changes in MHCII expression, antigen presentation, expression of accessory molecules, cytokine secretion, etc. [30]. However, effects of combined NOD2 and TLR4 stimulation using MDP and MPLA molecules absorbed on insoluble particles on DCs maturation was not investigated. To evaluate this, we incubated BMDCs with the vaccine formulations described above (Alum+OVA, Alum+OVA+MDP, Alum+OVA+MPLA and Alum+OVA+MDP+MPLA) for 18 hours and then used flow cytometry to measure the expression levels (indicated by MFI) of surface markers of DC maturation: MHC class II, CD80 and CD86 (Fig 4A, 4B and 4C). Treatment of BMDCs with formulations containing individual PRR agonists (Alum+OVA+MDP and Alum+OVA+MPLA) resulted in increased expression of CD80 (1.45- and 1.91-fold increases, respectively), CD86 (1.59- and 1.62-fold increases, respectively), MHCII molecules (5.21- and 4.78-fold increases, respectively) compared to untreated cells. However, the most significant enhancement of expression of maturation markers was detected after exposure of cells to the vaccine formulation containing both PRR ligands (Alum+OVA+MDP+MPLA), resulting in 2.66-fold, 2.39-fold, and 9.52-fold increases over the Alum+OVA formulation for CD80, CD86 and MHCII, respectively.

Activation of BMDCs was monitored by measuring cytokine production. BMDCs were incubated with the vaccine formulations described above for 24 hours and then the culture supernatants were collected, centrifuged to remove any cells, and assayed for levels of 23 different cytokines and chemokines using a multiplex bead-based kit. Addition to the cells vaccine formulations with adsorbed individual PRR agonists results in significant enhancement of cytokine production in comparison to vaccine particles containing no PRR agonists (Fig 4D). However, cytokine profiles were distinct in response to vaccine formulation. Addition of Alum+OVA+MDP vaccine formulation results in elevated levels of IL-1α (2.4-fold increase over untreated cells), IL-1β (2.0-fold increase), IL-2 (3.1-fold increase), IL-3 (2.8-fold increase), IL-6 (3.3-fold increase), IL-12 (p40) (2.5-fold increase), IL-13 (2.0-fold increase), GM-CSF (2.4-fold increase), MIP-1α (8.8-fold increase), MIP-1β (2.2-fold increase). Whereas, vaccine formulation containing MPLA (Alum+OVA+MPLA) results in enhanced production of IL-1α (13.0-fold increase over untreated cells), IL-1β (9.8-fold increase), IL-2 (4.6-fold increase), IL-3 (5.3-fold increase), IL-6 (2.9-fold increase), IL-10 (4.4-fold increase), IL-12 (p40) (1.7-fold increase), IL-12 (p70) (4.7-fold increase), IL-17a (8.8-fold increase), Eotaxin (3.0-fold increase), IFNγ (6.2-fold increase), KC (2.4-fold increase), TNFα (5.0-fold increase), MIP-1α (13.4-fold increase), MIP-1β (4.4-fold increase).

The most notable effect of cytokine production were detected in Alum+OVA+MDP+MPLA-treated cells. We found that vast majority of cytokines were significantly upregulated in comparison to cells treated with formulations containing individual PRR agonists (with the exception for IL-4, G-CSF, GM-CSF). Expression levels of 10 cytokines were “potentiated” (expression after combined stimulation of TLR4 and NOD2 relative to untreated cells was greater than the sum of the fold induction observed after stimulation of either TLR4 or NOD2 alone) after addition of vaccine formulation containing both TLR4 and NOD2 agonists: IL-1α (3.3-fold increase over the sum of the fold induction observed after stimulation of either TLR4 or NOD2 alone), IL-1β (4.2-fold increase), IL-2(4.2-fold increase) IL-10(4.2-fold increase) IL-12 (p70) (4.2-fold increase) (4.2-fold increase)IL-13(4.2-fold increase) IFN-γ(4.2-fold increase) KC(4.2-fold increase) TNFα (4.2-fold increase) MIP-1α (4.2-fold increase).

These results demonstrate that the synergistic effects of combined TLR4 and NOD2 stimulation in the context of alum-based vaccine formulations extend to promotion of BMDC maturation and activation.

### Vaccine formulations containing a combination of TLR4 and NOD2 agonists enhance the magnitude of cellular adaptive immune responses in vivo

**Fig 5:**
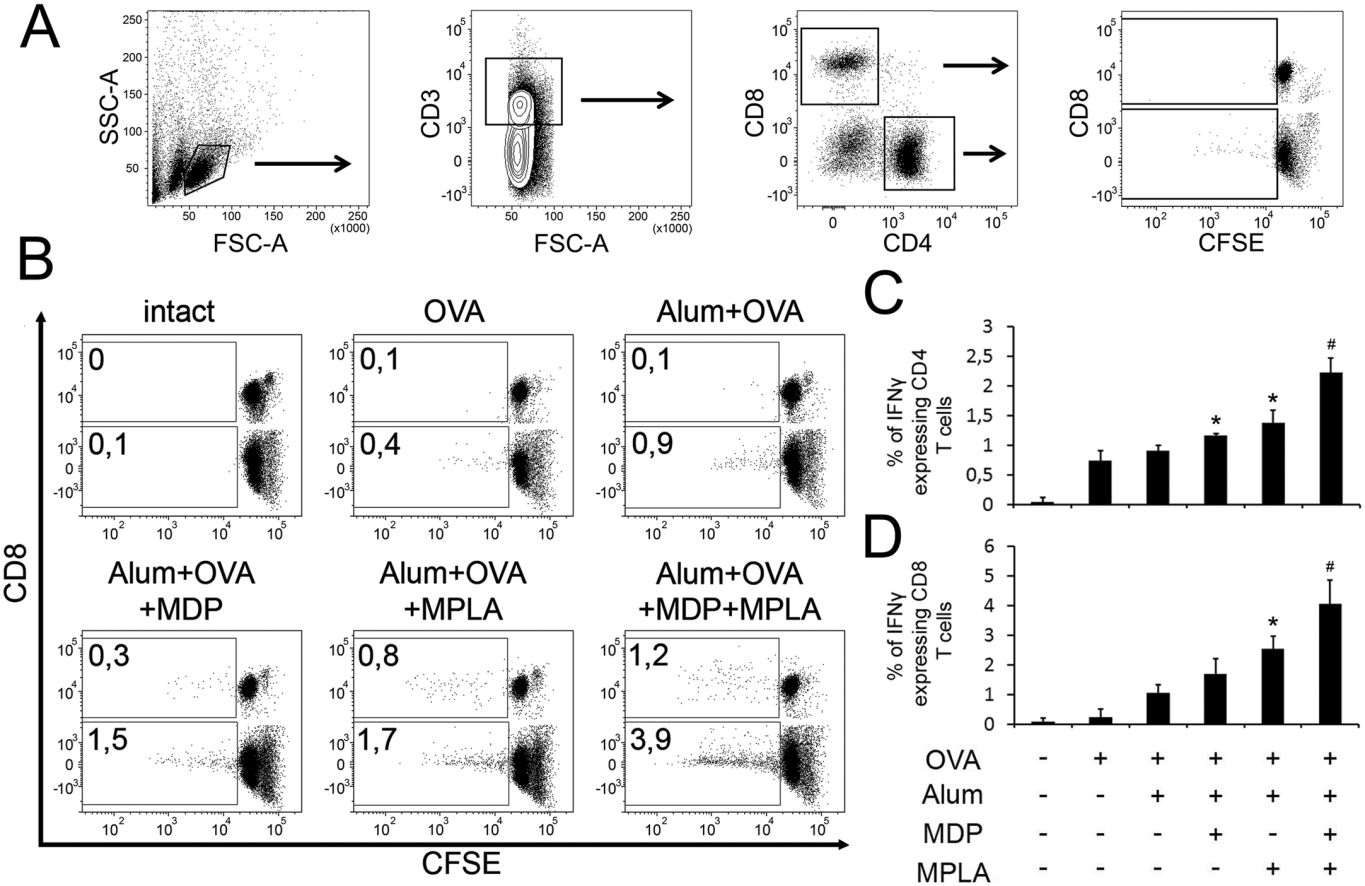
Vaccine formulations containing a combination of TLR4 and NOD2 agonists induce stronger antigen-specific CD4 and CD8 T-cell responses in mice than formulations containing individual PRR agonists. Mice (n = 5/group) were immunized s.c. twice with alum-based vaccine formulations: Alum+OVA, Alum+OVA+MDP, Alum+OVA+MPLA, and Alum+OVA+MDP+MPLA. Controls included naıve (untreated) mice and mice immunized with soluble OVA (no Alum or PRR agonist). Splenocytes were harvested from mice 14 days after the last immunization. **(A-B)** T-cell proliferation in response to ovalbumin restimulation. Splenocytes were CFSE-labeled, restimulated with whole OVA protein (1μg/ml) for 72h, stained with fluorescently-tagged antibodies against CD3, CD4 and CD8 and analyzed by flow cytometry. A. Gating strategy used for determination of antigen-specific CD4 and CD8 T-cell responses. B. Representative dot plots for the naïve (intact) group and each immunized group showing CFSE fluorescence (x-axis) of CD4 and CD8 T cells (distinguished by CD8 expression shown on the y-axis). The percentage of proliferating CD4+ and CD8+ T cells in response to antigen represent mean from two independent experiments with 5 mice/group each. **(C-D)** T-cell activation indicated by IFN-γ production in response to ovalbumin restimulation. Splenocytes were restimulated for 18 hours with whole OVA antigen at 1μg/ml in the presence of BD GolgiPlug solution (BD biosciences), stained with fluorescently-tagged antibodies against CD3, CD4 and CD8, permeabilized, stained with a fluorescently-tagged antibody against IFN-γ and then analyzed by flow cytometry. The percentage of CD4 (C) or CD8 (D) T cells expressing IFN-γ is shown as the mean ± SD for 5 mice per group. Two additional experiments yielded similar results * indicates significant difference (P≤0.05) between formulations containing MDP or MPLA individually and the formulation without PRR agonists (Alum+OVA). # indicates significant difference (P≤0.05) between Alum+OVA+MDP+MPLA treatment and Alum+OVA+MPLA or Alum+OVA+MDP (Student’s t-test).

The effects of including multiple PRR agonists in vaccine formulations on THP1 cells and BMDCs in vitro suggest that such formulations would be more effective vaccines in vivo than formulations containing only a single or no PRR agonist. To test this in terms of cell-mediated immunity, we used the formulations described above (Alum+OVA, Alum+OVA+MDP, Alum+OVA+MPLA and Alum+OVA+MDP+MPLA) as well as soluble OVA (no alum or PRR agonist) to immunize mice (two subcutaneous injections given two weeks apart, n = 5 mice/group) and then two weeks after the last immunization, splenocytes were isolated for analysis. T-cell responses were detected by CFSE fluorescence proliferation assay and intracellular IFNγ staining (72 and 18 hours after antigen restimulation in vitro, respectively). Gating strategy for determination of antigen-specific CD4+ and CD8+ T-cell proliferative responses presented in Fig 5A. FACS plots from a one representative experiment in each group is shown (Fig 5B). The mean proliferative response of OVA-restimulated splenic T cells isolated from five mice immunized with soluble OVA or OVA absorbed on alum particles was essentially limited to CD4 T cells (0.4% and 0.9%, respectively; compared to 0.1% for untreated mice). In contrast, adsorption of PRR agonists along with OVA on alum particles resulted in increased proliferation within both the CD4 and CD8 T cell populations after in vitro OVA restimulation. Compared to Alum+OVA-immunized mice those immunized with Alum+OVA+MDP or Alum+OVA+MPLA had additional 0.6% CD4+, 0.2% CD8+ T cells and 0.8% CD4+, 0.7% CD8+ T cells proliferated, respectively. The most robust lymphoproliferative response was observed in the mice immunized with Alum+OVA+MDP+MPLA: additional 3.0% CD4+ T cells and 1.1% CD8+ T cells proliferated in comparison to Alum+OVA group.

Consistent with the T cell proliferative response detected by CFSE staining, addition of PRR agonists to vaccine formulations promoted T cell activation as indicated by an increase in IFN-γ production by CD4 and CD8 T cells 18 h after in vitro OVA-restimulation of splenocytes from immunized mice (Fig 5C). Statistically significant increases in the numbers of IFN-γ-positive T cells were seen with Alum-OVA-MDP and Alum-OVA-MPLA immunization (1.2% of CD4+ T cells, 1.7% of CD8+ T cells, and 1.4% of CD4+ T cells, 2.6% of CD8+ T cells, respectively) compared to 0.9% and 1.1% with Alum-OVA immunization. The most intensive response were observed in Alum+OVA+MDP+MPLA-immunized group (2.2% of CD4 T cells and 4.1% of CD8 T cells).

Thus, immunization of mice with the Alum+OVA+MDP+MPLA vaccine formulation resulted in an enhanced T cell response compared to immunization with vaccine formulations without PRR agonists or containing individual PRR agonists. This strongly supports the concept of improved immunoadjuvant efficacy based on the synergistic effects of combined activation of PRRs from multiple families.

### Vaccine formulations containing a combination of TLR4 and NOD2 agonists enhance the magnitude of humoral adaptive immune responses

To evaluate the humoral adaptive immune response induced by vaccine formulations containing individual or combined PRR agonists, we immunized mice as described above (two subcutaneous injections given two weeks apart, n = 5 mice/group) with soluble OVA (no alum or PRR agonist), Alum+OVA, Alum+OVA+MDP, Alum+OVA+MPLA or Alum+OVA+MDP+MPLA formulations and collected blood 14 days after the last immunization for determination of serum titers of total IgG and different IgG isotypes.

**Fig 6:**
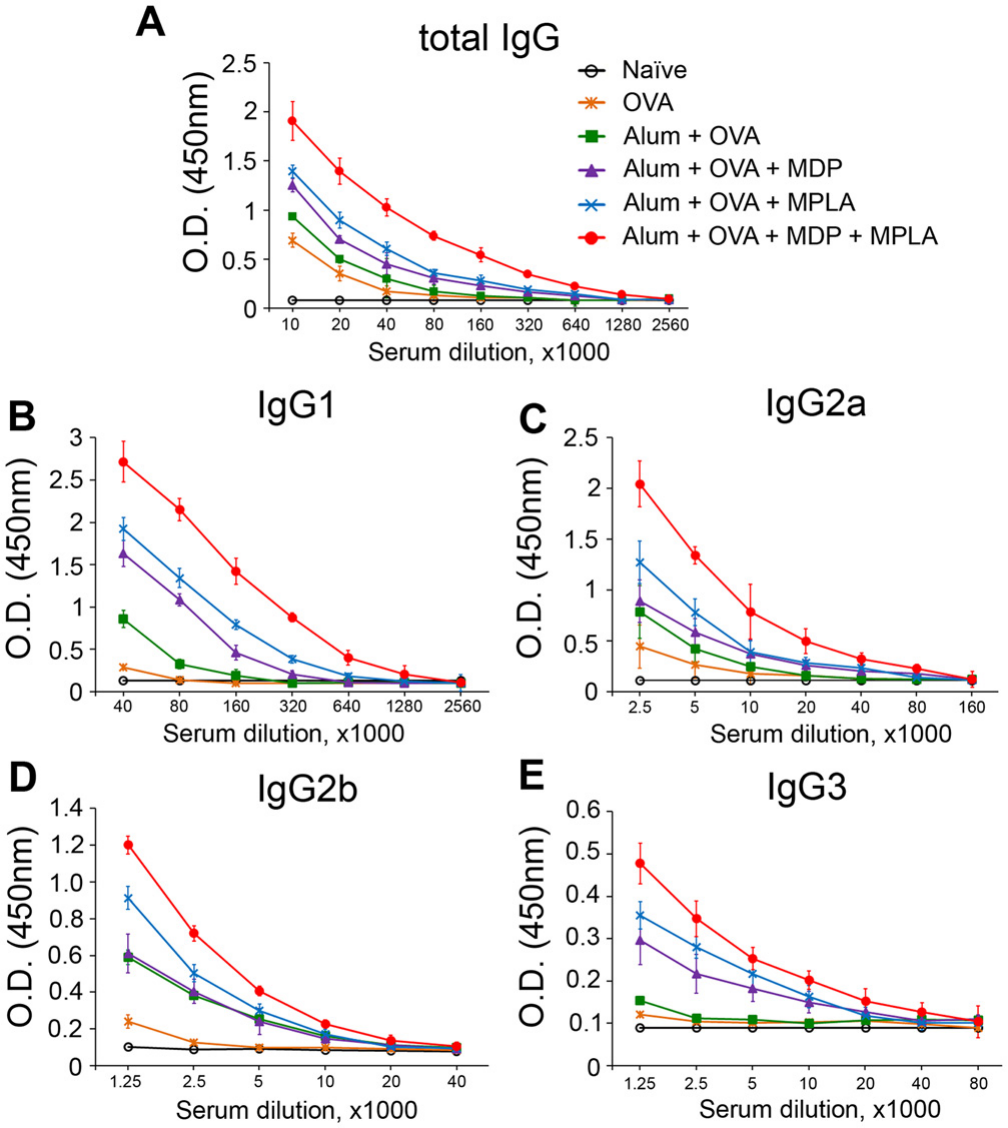
Vaccine formulations containing a combination of TLR4 and NOD2 agonists lead to a stronger ovalbumin-specific antibody response in mice than formulations containing individual PRR agonists. Mice (n = 5/group) were immunized s.c. twice with alum-based vaccine formulations: Alum+OVA, Alum+OVA+MDP, Alum+OVA+MPLA, and Alum+OVA+MDP+MPLA. Controls included naıve (untreated) mice and mice immunized with soluble OVA (no Alum or PRR agonist). Blood was collected from mice 14 days after the last immunization and serum levels of ovalbumin-specific total IgG (A), IgG1 (B), IgG2a (C), IgG2b (D) and IgG2c (E) antibodies were detected by ELISA. The mean for 5 mice/group ± SEM is shown. Experiment was repeated three times with analogous results.

Immunization of mice with vaccine formulations containing individual PRR agonists (Alum-OVA-MDP or Alum-OVA-MPLA) resulted in production of higher levels of total ova-specific IgG antibodies than immunization with the Alum-OVA formulation without any PRR agonist (mean IgG titer: 320,000, 160,000 and 80,000, respectively) (Fig 6A). The formulation containing both PRR agonists (Alum+OVA+MDP+MPLA) was even more immunogenic, as indicated by a total ova-specific IgG titer of 640,000, which was 2-fold higher than that seen with the Alum+OVA+MPLA formulation and 4-fold higher than that seen with the control Alum+OVA formulation without PRR agonists. Physical adsorption of the ovalbumin antigen on alum particles (no PRR agoinst; Alum+OVA) resulted in a little less than a 2-fold increase in total ova-specific IgG titer compared to soluble ovalbumin.

Further analysis revealed that the vaccine formulation containing both MDP and MPLA induced higher levels of ova-specific antibodies of all IgG subtypes (IgG1, IgG2a, IgG2b, IgG3) in comparison to Alum-OVA-MDP and Alum-OVA-MPLA formulations (Fig 6B, 6C, 6D and 6E). Interestingly, the Alum-OVA-MPLA formulation induced 2-fold higher titers of IgG1 subtype antibodies than the Alum-OVA-MDP formulation, whereas differences in IgG2a, IgG2b, and IgG3 titers between these groups were not significant. This observation could have probable explanation basing on the fact that engagement of TLR4 but not NOD2 results in MyD88-dependent activation of NF-κB, whereas IgG1 response is dependent on the expression of MyD88 in B cells [31].

Thus, obtained results indicate that combined stimulation of TLR4 and NOD2 receptors in the context of alum-based ovalbumin vaccination of mice enhances the magnitude of anti-ovalbumin humoral immune responses compared to stimulation of only one PRR family member.

## Discussion

Vaccination is a highly effective biomedical approach to controlling infectious diseases. The goal of vaccination is to stimulate generation of a strong and durable immune response against the administered antigen, providing long-term protection against infection. However, use of highly purified recombinant or synthetic antigens in subunit vaccines often makes them generally less immunogenic than vaccines containing live or killed whole organisms. Recent data indicates that this may be due to the fact that recombinant/synthetic vaccines often lack markers of pathogenicity (‘danger’ signals) that are part of the infectious organism and are essential for activation of full-bodied innate immune responses that contribute to development of adaptive immunity. A significant breakthrough in our understanding of how the immune system recognizes and generates protection against pathogenic microbial organisms resulted from the discovery of pattern recognition receptors (PRRs) and the associated signaling pathways [32,33,34]. It was determined that PRRs recognize evolutionarily conserved molecular components of pathogens, termed pathogen associated molecular patterns (PAMPs). PRR–PAMP interactions trigger intracellular signaling cascades that culminate in expression of a variety of proinflammatory molecules, which together orchestrate the early innate immune response to infection and also affect subsequent activation of adaptive immunity [35].

These findings presented new opportunities for vaccine design, and the concept of ‘danger’ signals has been successfully applied to develop modern adjuvants that assist antigens in achieving high efficacy and safety parameters for vaccines by enhancing and directing the immune response [4]. Thus, currently, it is widely believed that an “ideal” adjuvant should mediate its activity through several mechanisms, including: (i) presenting ‘danger’ signals that are able to trigger PRRs that activate innate immune responses; (ii) acting as an effective antigen delivery system resulting in improved antigen uptake by APCs and lymphatic trafficking; (iii) promoting localized immune activation and inducing production of proinflammatory cytokines for immune cell recruitment [15].

While the beneficial effect of PRR stimulation on vaccine efficacy has been established, the potential for even greater adjuvant activity with simultaneous stimulation of multiple PRR families is still understudied. The rationale for this approach includes growing evidence showing that during any infectious event, various types of PRRs are activated in a stepwise manner [36]. This suggests importance of complementary as well as synergistic effects of signaling via PRRs belonging to different families for efficient development of protective immunity against a given pathogen. Additional support for this thesis is provided by data showing synergistic action between members of different families of PRRs (e.g., Toll-like and Nod-like receptors) leading to enhanced levels of transcription factor activity and cytokine/chemokine production, and ultimately, increased protection of mice against Salmonella infection in comparison to stimulation of just one type of PRR [18,19,20]. Based on this foundation, the goal of the work reported here was to determine whether cooperation/synergy between different PRR families affects adaptive immunity and could be applied towards improving the efficacy of vaccination.

To address this goal, we created vaccine formulations containing alum particles (as the particulate component of the vaccine) with a model antigen (ovalbumin) and the TLR4 agonist MPLA and the NOD2 agonist MDP adsorbed to the alum particles separately or in combination. We found that compared to use of individual PRR agonists, combining both PRR agonists within the vaccine formulation synergistically enhanced activation of transcription factors NF-κB and AP-1 and production of proinflammatory cytokines in THP-1 cells. These observations are consistent with previous reports using soluble PRR agonists while additionally demonstrating that absorption of MPLA and MDP on a particulate carrier does not affect synergism between TLR4 and NOD2 signaling.

One of the first key steps in the induction of immune responses is internalization of the antigen by antigen presenting cells, such as dendritic cells (DCs). It was previously shown that stimulation of TLR4 and NOD2 play a crucial role in upregulation of the process of antigen phagocytosis by DCs [37,38], but potential synergy upon combined stimulation was not evaluated. Our data indicate that, indeed, combined stimulation of both NOD2 and TLR4 in the context of an alum-based vaccine formulation results in enhanced levels of antigen uptake by BMDCs compared to stimulation of only one PRR or the other. While the mechanism underlying the synergistic effect of PRR stimulation on vaccine particle uptake by DCs remains to be investigated, several TLR-dependent mechanisms of bacteria and vaccine particle uptake are well documented [39]. Based on our results, we speculate that TLR and NOD receptor pathways possibly have an essential common intracellular signaling point through which synergy is generated.

Another critical step for further induction of T lymphocyte-dependent immunity is maturation and activation of DCs [40]. Earlier studies demonstrated upregulation of DC maturation markers (CD80, CD86, etc.) and cytokine production in response to stimulation of TLR4 or NOD2 individually [41,42]. However, since virtually all pathogens contain molecules that may be detected by PRRs belonging to different families [36], the effects of combined stimulation of different types of PRRs on DC activation are more biologically relevant. Here, we demonstrated that in vitro treatment of immature BMDCs with vaccine formulations containing agonists of either TLR4 (Alum-OVA-MPLA) or NOD2 (Alum-OVA-MDP) resulted in increased expression of the DC maturation/activation markers MHCII, CD80, and CD86, but that the vaccine formulation with both PRR agonists (Alum-OVA-MDP-MPLA) had a significantly stronger effect. Increased expression of CD80/86 in DCs is known to be critical for antigen-specific activation of Th cells [43]. In addition, optimal activation of DCs favors local activation of Th cells expressing TCRs with the highest affinity to antigenic peptides [44]. The Alum-OVA-MDP-MPLA vaccine formulation had a similar statistically significant synergistic effect (compared to Alum-OVA-MDP or Alum-OVA-MPLA) on BMDC production of cytokines and chemokines, some of which are known to be important for CD4 T cell-DC interaction (e.g., MIP-1α, MIP-1β) [45] or in serving as chemoattractants for NK cells, neutrophils and macrophages (e.g., MIP-1α, MIP-1β, KC, RANTES) [46]. We also observed that combined stimulation of TLR4 and NOD2 also notably enhanced production of number of cytokines belonging to Th1-polarizing (IL-2, TNF, IFN-γ) as well as Th2-polarazing groups (IL-5, IL-6, IL-10, IL-13) [47].

Therefore, the results of these analyses suggest that combined stimulation of TLR4 and NOD2 is likely to promote mixed Th1/2-mediated immunity. Consistent with this, when the Alum-OVA-MDP, Alum-OVA-MPLA and Alum-OVA-MDP-MPLA vaccine formulations were used to immunize mice, enhanced cellular and humoral immune responses were observed with stimulation of combined, as opposed to individual, PRRs. The synergistic effect of combined PRR stimulation in vivo on cellular immunity was indicated by a significant increase in the proportion of splenic CD4 and CD8 T cells that were proliferating and/or activated (IFN-γ producing) after in vitro restimulation with ovalbumin. The enhanced humoral response was indicated by higher titers of ovalbumin-specific IgG antibodies in mouse serum samples, including all four IgG sybtypes relevant to a mixed Th1/Th2 response [48]. Therefore, vaccines including combined PRR agonists induce significantly stronger humoral and cellular immune responses to a model antigen in vivo without significant Th1/Th2 polarization.

In summary, this is the first report showing that combined stimulation of two distinct PRR families (Toll- and NOD-like receptors) using MPLA and MDP molecules formulated into an alum-based vaccine effects on several critical steps of stimulation of immune response: significantly enhances antigen uptake by BMDCs, promotes BMDC maturation and activation and leads to enhanced humoral and cellular adaptive immune responses in comparison to alum adjuvant alone or alum particles with absorbed individual PRR agonists. Our results show that collaboration between members of different families of PRRs is not limited to regulation of innate immunity, but also affects the magnitude of subsequent adaptive immune responses. Overall, this study provides strong proof of concept for development of a new class of adjuvants based on combined PRR stimulation that could significantly improve the efficacy of vaccines. It may even be possible to tailor such adjuvants to specific infectious agents by using optimal compositions of PRR agonists to induce a desired type of adaptive immunity (e.g., Th1, Th2, or Th17).

## Supporting Information

S1 Fig Combined stimulation of TLR4 and NOD2 receptors leads to enhanced NF-κB/AP-1-dependent SEAP activity in THP1-XBlue^™^-CD14 cells. SEAP activity resulting from NF-κB/AP-1-dependent SEAP reporter gene expression was measured in THP1-Xblue^™^-CD14 cells 18 h after treatment with the indicated doses (μg/ml) of MPLA and MDP alone or in combination. Results are expressed as the fold increase in SEAP activity relative to intact (untreated) cells. The values presented are the mean fold-increase from three independent experiments with duplicate samples in each experiment. Error bars indicate the SD.
(TIF)

S2 Fig Combined stimulation of NOD2 and TLR4 receptors leads to enhanced cytokine production in THP1 cells. Combined stimulation of NOD2 and TLR4 receptors leads to enhanced cytokine production in THP1 cells. Cells were left untreated or treated with MDP (20 μg/ml), MPLA (1 μg/ml), or their combination for 18 hrs. Cell-free supernatants were prepared and analyzed by multiplex-bead ELISA Bio-Plex Pro kit (BioRad, USA) for production of IL-1β, TNF-α, and IL-8. The values shown are the mean ± SD from triplicate wells. Results are representative of at least three separate experiments.
(TIF)

S3 Fig The stability of vaccine formulations depends on the dose of ovalbumin absorbed on alum particles. Depletion of the zeta potential (A) of alum particles using higher doses of ovalbumin results in particle aggregation, which corresponds to an increase in the mean diameter of particulates (B). The values shown are the mean ± SD for three batches of Alum+OVA vaccine formulation generated with each indicated ovalbumin dose.
(TIF)

S1 Table Physico-chemical characteristics of alum-based vaccine formulations. Particle size, polydispersity index (PDI) and zeta-potential of alum-based vaccine formulations Alum (n = 3), Alum + ova (n = 3), Alum + ova + MDP (n = 3), Alum + ova + MPLA (n = 3), Alum + ova + MDP+MPLA (n = 3). Results are expressed as mean ± standard deviation (SD).
(DOCX)
