# Supplementary figures and images for: Powerful Complex Immunoadjuvant Based on Synergistic Effect of Combined TLR4 and NOD2 Activation Significantly Enhances Magnitude of Humoral and Cellular Adaptive Immune Responses
Source: PLoS One. 2016 May 17;11(5):e0155650. doi: 10.1371/journal.pone.0155650 (PMC4871337; doi:10.1371/journal.pone.0155650)

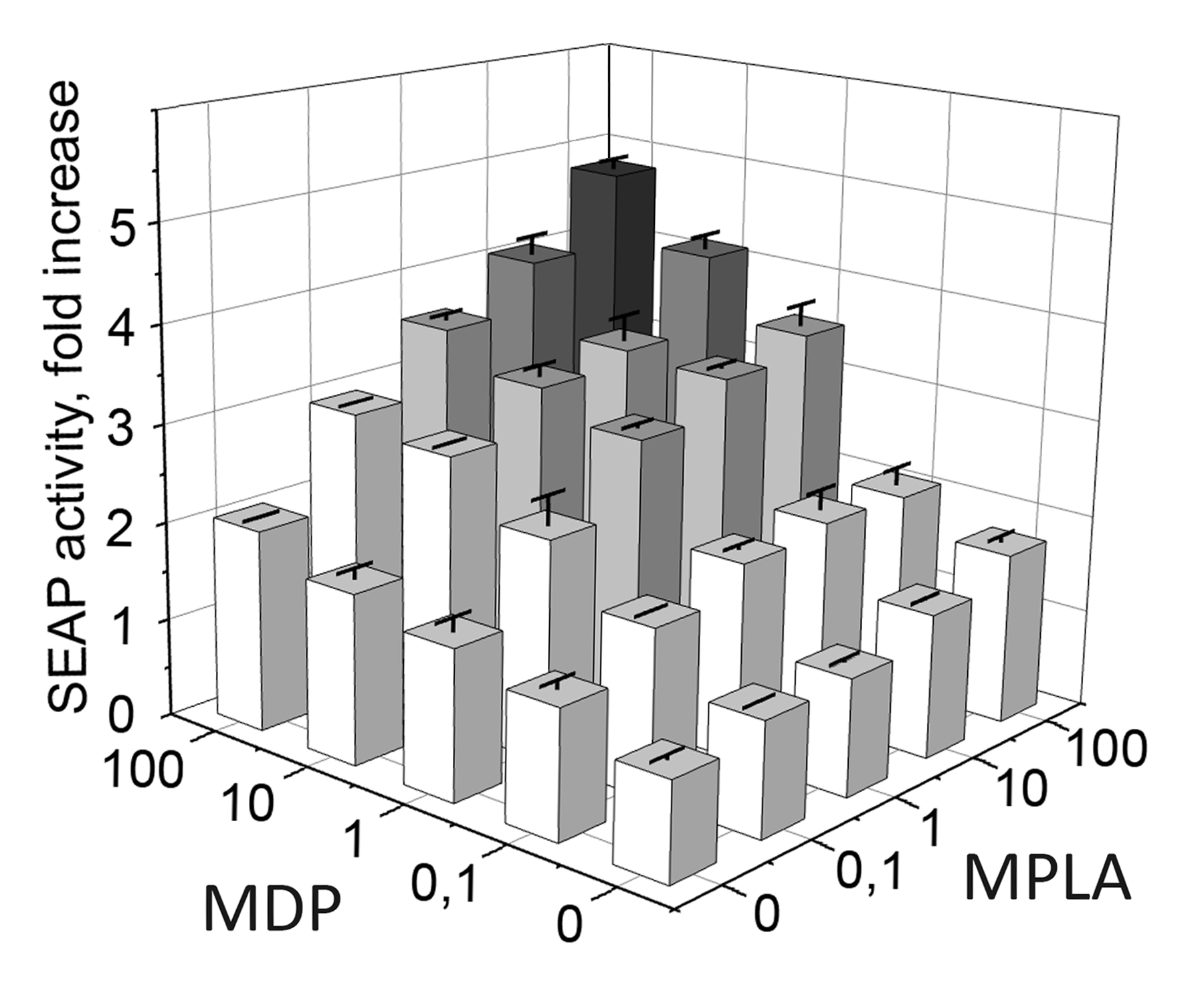

Supplement: S1 Fig — SEAP activity resulting from NF-κB/AP-1-dependent SEAP reporter gene expression was measured in THP1-Xblue™-CD14 cells 18 h after treatment with the indicated doses (μg/ml) of MPLA and MDP alone or in combination. Results are expressed as the fold increase in SEAP activity relative to intact (untreated) cells. The values presented are the mean fold-increase from three independent experiments with duplicate samples in each experiment. Error bars indicate the SD. (TIF) [file pone.0155650.s001.tif]

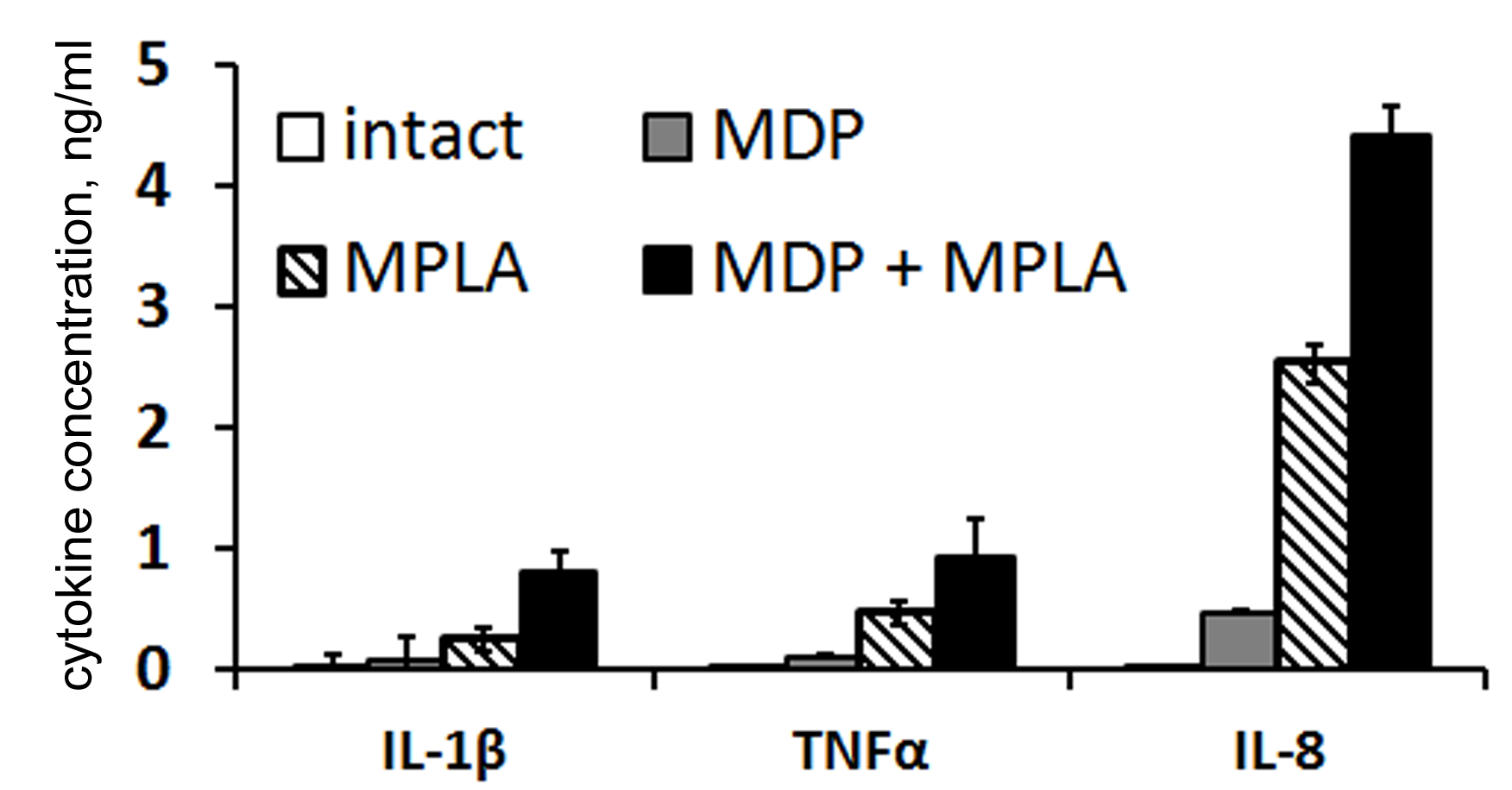

Supplement: S2 Fig — Combined stimulation of NOD2 and TLR4 receptors leads to enhanced cytokine production in THP1 cells. Cells were left untreated or treated with MDP (20 μg/ml), MPLA (1 μg/ml), or their combination for 18 hrs. Cell-free supernatants were prepared and analyzed by multiplex-bead ELISA Bio-Plex Pro kit (BioRad, USA) for production of IL-1β, TNF-α, and IL-8. The values shown are the mean ± SD from triplicate wells. Results are representative of at least three separate experiments. (TIF) [file pone.0155650.s002.tif]

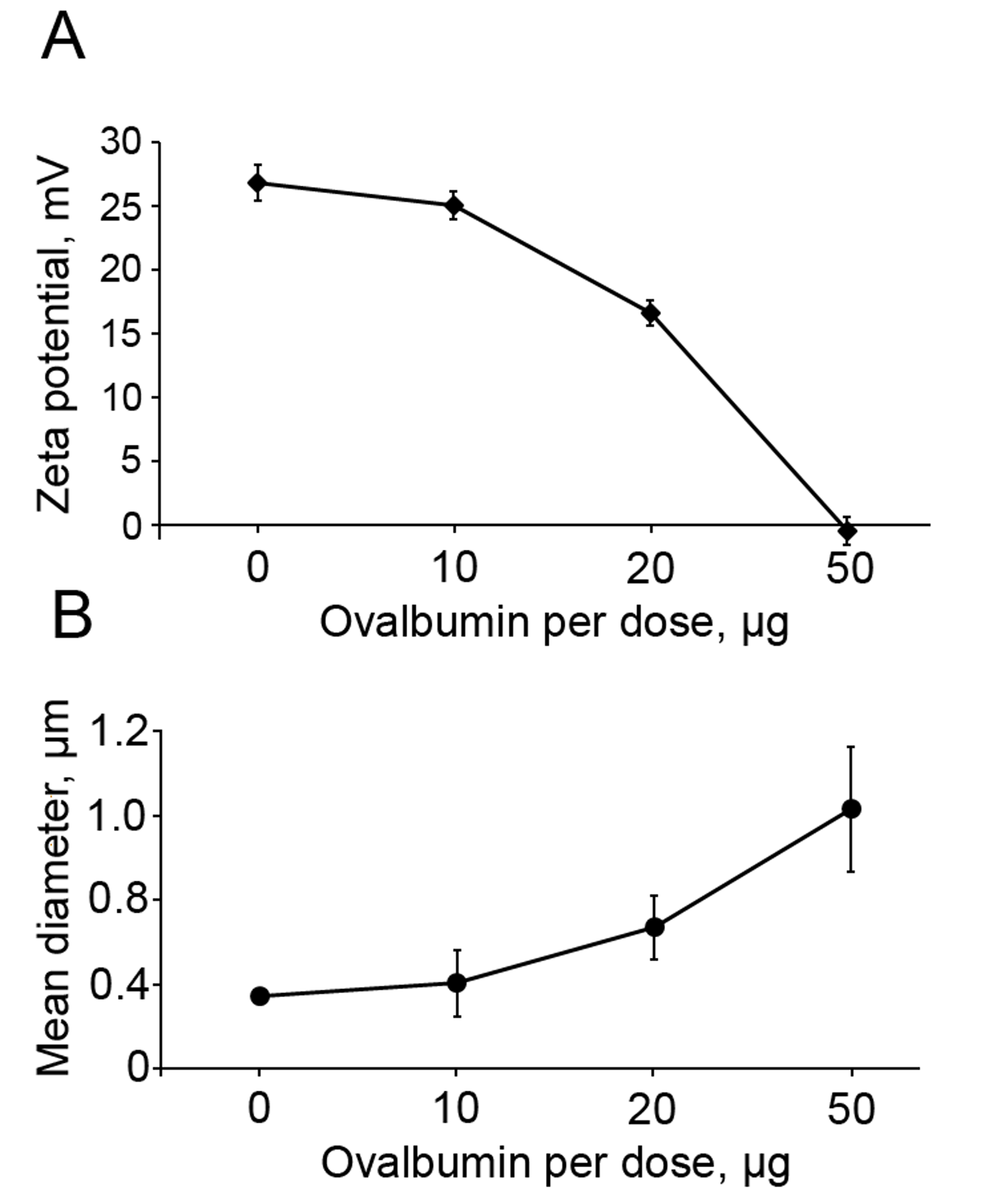

Supplement: S3 Fig — Depletion of the zeta potential (A) of alum particles using higher doses of ovalbumin results in particle aggregation, which corresponds to an increase in the mean diameter of particulates (B). The values shown are the mean ± SD for three batches of Alum+OVA vaccine formulation generated with each indicated ovalbumin dose. (TIF) [file pone.0155650.s003.tif]
