# Supplementary material for: Powerful Complex Immunoadjuvant Based on Synergistic Effect of Combined TLR4 and NOD2 Activation Significantly Enhances Magnitude of Humoral and Cellular Adaptive Immune Responses
Source: PLoS One. 2016 May 17;11(5):e0155650. doi: 10.1371/journal.pone.0155650 (PMC4871337; doi:10.1371/journal.pone.0155650)
Supplement: S1 Table — Particle size, polydispersity index (PDI) and zeta-potential of alum-based vaccine formulations Alum (n = 3), Alum + ova (n = 3), Alum + ova + MDP (n = 3), Alum + ova + MPLA (n = 3), Alum + ova + MDP+MPLA (n = 3). Results are expressed as mean ± standard deviation (SD). (DOCX) [file pone.0155650.s004.docx]

| Vaccine type | Z-Average (d.nm) | Polydispersity index (PDI) | Zeta potential (mV) | Absorption efficacy, % | | |
| --- | --- | --- | --- | --- | --- | --- |
|  |  |  |  | Ovalbumin | MDP | MPLA |
| Alum | 643,0 ± 22,6 | 0,438 ± 0,156 | + 26,8±1,42 | - | - | - |
| Alum + ova | 706 ± 53,9 | 0,657 ± 0,225 | + 25,0±1,14 | 98,9±0,4 | - | - |
| Alum + ova + MPLA | 769 ± 88,17 | 0,812 ± 0,040 | + 21,1±1,43 | 98,6±0,2 | - | 93,4±0,9 |
| Alum + ova + MDP | 764 ± 71,8 | 0,713 ± 0,177 | + 23,6±1,08 | 98,7±0,3 | 99,2±0,1 | - |
| Alum + ova +  MDP + MPLA | 839,0 ± 128,54 | 0,753 ± 0,128 | +18,02±1,17 | 98,4±0,2 | 99,1±0,1 | 90,7±0,4 |
